# Effect of Aging Time on Microstructure and Mechanical Properties in a Cu-Bearing Marine Engineering Steel

**DOI:** 10.3390/ma13163638

**Published:** 2020-08-17

**Authors:** Mingxue Sun, Yang Xu, Jin Wang

**Affiliations:** School of Mechanical and Automotive Engineering, Qingdao University of Technology, Qingdao 266520, China; xuyang_steel@163.com (Y.X.); wangjin@qut.edu.cn (J.W.)

**Keywords:** Cu-bearing marine engineering steel, Cu precipitation, aging time, microstructure, mechanical property

## Abstract

This study elucidated the structure–property relationship in a Cu-bearing marine engineering steel that was water cooled and then aged at 500 °C for 0.5–3 h. The microstructural features, tensile properties and impact properties were comparatively investigated as a function of aging time. When the aging period was increased, the Cu precipitates underwent coarsening, and a stable face-centered cubic (fcc) formation occurred. Additionally, the tensile properties were significantly improved at the expense of low temperature toughness, which can be attributed to the presence of nano-sized Cu precipitates. The increment of yield strength mainly derived from Cu precipitate–dislocation interaction strengthening effects (232 MPa for 1 h and 199 MPa for 3 h.) during aging process. Therefore, optimization of mechanical properties was achieved by controlling the parameters of aging process. The peak age hardening condition (i.e., at 500 °C for 1 h) resulted in the yield strength of 755 MPa, tensile strength of 812 MPa, elongation of 26.3% and impact energy of 78 J at −80 °C.

## 1. Introduction

Cu-bearing high strength low alloy (HSLA) steels, which possess a desirable combination of strength, ductility, weldability and toughness, have drawn much attention in the construction of naval warships, offshore drilling platforms, oil pipelines and bridges [1,2,3,4]. In recent years, there have been great efforts for improving the weldability of these materials [5,6]. To achieve good weldability and high impact toughness, the carbon content is kept below 0.08 wt% in Cu-bearing HSLA steels. The addition of Cu can strengthen the matrix via the formation of nano-sized Cu precipitates without diminishing the weldability. Since Cu serves as the main strengthening element during the manufacturing process, the understanding of Cu precipitation behaviors has been a major choice to develop these steels [7,8,9,10].

Many research papers have been published on the quenching and aging conditions required for the development of Cu-bearing HSLA steels [11,12,13,14]. Dhua et al. [11] investigated the effects of tempering temperature on the mechanical properties and microstructures of HSLA-100 type Cu-bearing steels. They noted that coarsening of Cu precipitates occurred when the tempering temperature was higher than 500 °C. Hwang et al. [12] utilized the direct quenching and tempering (DQ&T) process in low-carbon Cu-bearing steels to establish a relationship between the microstructure and mechanical properties.

For Cu-bearing HSLA steels, bainitic and martensitic structures can be obtained during quenching process due to the high hardenability. Structure recovery during aging treatment can provide an improvement on impact toughness [11,12,13,14,15,16]. Dhua et al. [11] reported that a mixture of martensite, bainitic ferrite and retained austenite can be obtained in water-quenched condition. During aging process, the evolution of mechanical properties was determined by the recovery of matrix and formation and coarsening of Cu precipitates. Ghosh et al. [14] described that the microstructure consisted of acicular ferrite and bainitic ferrite in thermo-mechanical control process (TMCP). Coherent body-centered cubic (bcc) Cu precipitates provided peak hardness at aging temperature of 425 °C, while continuous recovery of matrix, annihilation of dislocation and coarsening of Cu precipitates caused a continuous decrease in hardness at aging temperature above 450 °C. Despite extensive research on the above-mentioned forms of Cu-bearing HSLA steels, there are few studies focused on the ferritic structure. Ghosh et al. [3] studied the effect of cooling rate on structure–property relationship of an ultra low carbon HSLA-100 steel. The microstructure transformed from aciular ferrite to polygonal or quasi-polygonal ferrite with the decrease of cooling rate, which was accompanied by the dropping of strength and improvement of ductility. Liu et al. [17] found that at cooling rate of 0.1 °C/s, interphase precipitation occurred along with the formation of polygonal ferrite, while Cu precipitates cannot be observed in acicular ferrite, retained austenite and martensite. It can be seen that these reports are mainly concentrated in Cu precipitation behaviors in ferritic structure during continuous cooling process, while the formation of Cu precipitates in ferrite is quite different from aging treatment. Mulholland et al. [9] reported the nanoscale co-precipitation of a high strength low carbon steel treated by DQ&T process. Coarsening of Cu precipitates can be counterbalanced by the nucleation and growth of carbide precipitates, leading to the maintenance of high yield strength for as long as 320 h at aging time of 450 °C. The dissolution of Fe_3_C contributed to an increase in impact toughness after aging at 450 °C for 80 h. Previous studies mainly achieved Cu precipitates utilizing DQ&T process, resulting in a higher production cost. With suitable composition design, fine ferritic structure that exhibits good low temperature toughness and ductility can be obtained by use of TMCP with ultra-fast cooling (UFC) equipment. By taking advantage of the benefits inferred through Cu precipitation during the subsequent aging treatment, the ferrite matrix can be significantly strengthened using TMCP&T process.

In the current study, the effects of aging time on the microstructural features and mechanical properties of a Cu-bearing marine engineering steel were investigated using scanning electron microscope (SEM) (Jena, Germany), transmission electron microscope (TEM) (Hillsboro, United States), tensile tests and Charpy impact analysis. The precipitation and coarsening behavior of Cu and their impact on mechanical properties were discussed in detail. The effects of aging time on microstructural evolution, Cu precipitation behaviors and mechanical properties were systematically studied. It can be concluded that Cu precipitate–dislocation interaction strengthening was the decisive factor for the strength improvement. The outstanding combination of strength, elongation and impact energy can be achieved at aging temperature of 500 °C for 1 h.

## 2. Materials and Methods

**Figure 1:**
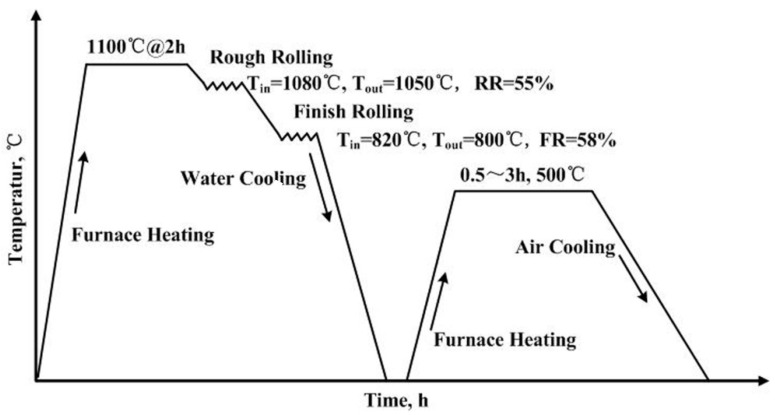
Schematic of the processing methods used in this study.

An ultra low carbon steel with a composition of 2.0 wt% Cu and 2.5 wt% Ni was prepared, and the concrete chemical composition of the steel is provided in Table 1. The steel was melted in a vacuum induction furnace and cast into billets. The ingots were subsequently heated to 1100 °C for 2 h and hot rolled into 15-mm-thick plates in a two-high 450-mm pilot mill. As shown in Figure 1, the rolling process was conducted in two stages. The first stage, aptly named “Rough rolling”, was conducted between 1080 (T_in_) and 1050 °C (T_out_). The temperature of the “Finish rolling” stage was between 820 (T_in_) and 800 °C (T_out_). The corresponding rough rolling reduction (RR) and finish rolling reduction (FR) were 55% and 58%, respectively. The reduction schedule was 80 → 54 → 36 → 29 → 23 → 19 → 15 (mm). After rolling, the steel slabs were water cooled to room temperature at a cooling rate of 20 °C/s, followed by aging at 500 ℃ for 0.5, 1, 1.5 and 3 h.

Microstructural analysis was determined using an ULTR A^TM^ 55 SEM and a FEI Tecnai G^2^ F20 TEM. SEM samples were prepared and etched with 2% nital solution. The samples for TEM observation were conducted by cutting thin slices and burnishing them using sandpapers, followed by electropolishing in an electrolyte mixture containing 87.5% ethyl alcohol and 12.5% perchloric acid. To evaluate the mechanical properties of the tested steel, tensile tests and Charpy impact tests were conducted using a CMT-5105 testing machine and a JBW-500 impact testing machine, respectively. The tensile samples, which were produced according to ASTM E8M guidelines, were 8 mm in diameter and 40 mm in gauge length. Standard specimens (10 mm × 10 mm × 55 mm) were prepared for the Charpy impact tests, consistent with ASTM E23 standard. The impact tests were performed at −40, −60 and −80 °C. Clear fracture mode results were obtained by investigating the fracture surfaces using SEM.

## 3. Results and Discussion

### 3.1. Microstructural Characterization by SEM

**Figure 2:**
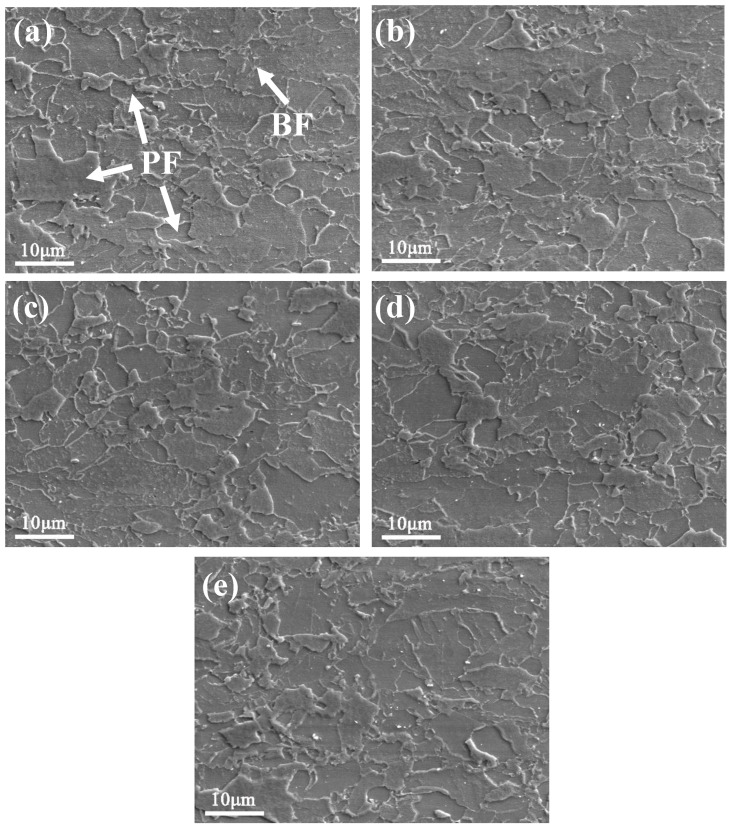
SEM images of the tested steel samples: (**a**) after water cooling; and after aging at 500 °C for (**b**) 0.5 h; (**c**) 1 h; (**d**) 1.5 h; and (**e**) 3 h.

Figure 2 shows the SEM images of the tested steel samples. Polygonal ferrite (PF) can be observed in water-cooled condition, which appears equiaxed and polyhedral in shape. Using a linear interception method, the average size of the ferrite grain was measured to be ~5.4 µm. In addition, a small amount of bainitic ferrite (BF) structure, which is characterized by the presence of lath bainite morphology, was also observed. As compared to water-cooled condition, the structures remained stable with the increase of the aging time, as shown in Figure 2b–e. TEM analysis of the refined microstructures is discussed in subsequent sections.

### 3.2. Microstructural Characterization by TEM

**Figure 3:**
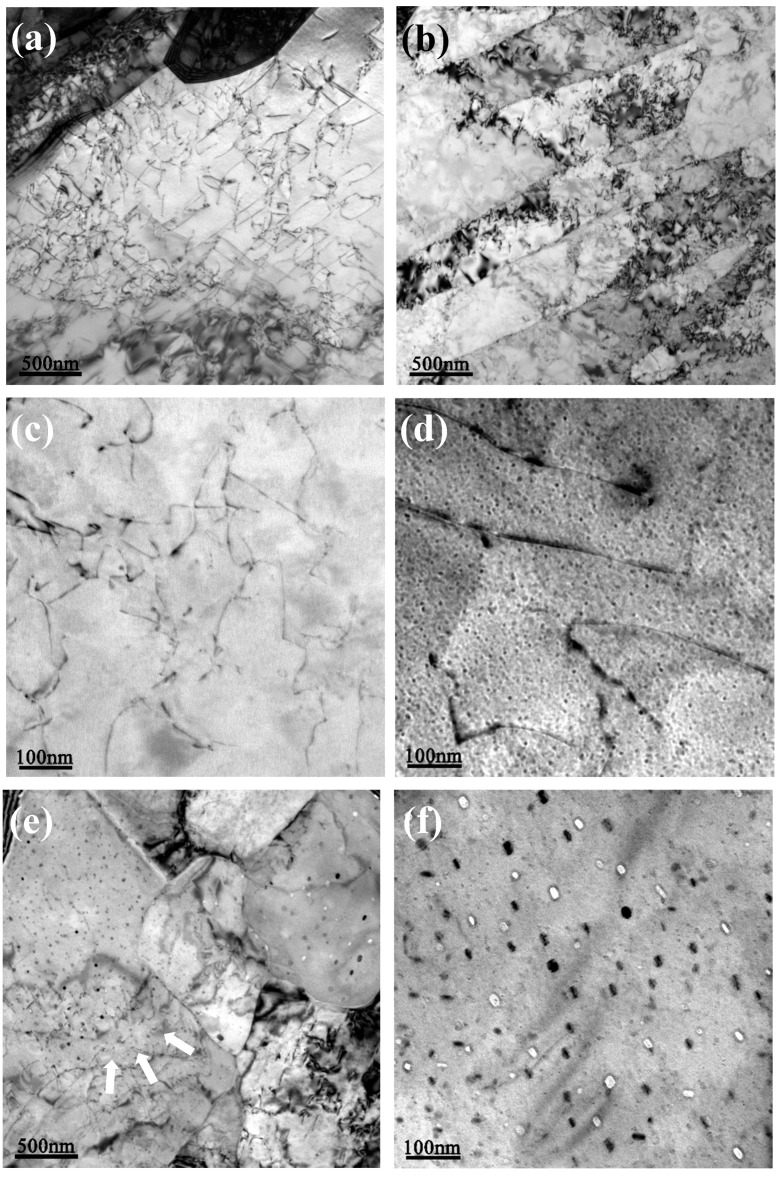
Bright-field TEM images of the tested steel samples for: (**a**) high density dislocations after water cooling; (**b**) bainitic ferrite laths after water cooling; (**c**) low density dislocations after aging for 0.5 h; (**d**) fine Cu precipitates after aging for 1 h; (**e**) recovery of the matrix showing the annihilation of dislocations and growth of Cu precipitates after aging for 3 h; and (**f**) coarsened Cu precipitates after aging for 3 h.

Figure 3 shows bright-field TEM images of the tested steel samples for the various processing methods. Five images were used to determine the average size of precipitates using Image-Pro Plus software. As shown in Figure 3a, the high-density dislocations observed within the ferrite grains were produced in the fast cooling region. In addition, the lath-like structures were found to have an average width of 0.6 μm. During isothermal aging at 500 °C for 0.5 h, the dislocation density decreases slightly due to the annihilation of dislocations. Cu precipitates were challenging to observe in the resulting TEM image, as shown in Figure 3c. However, when the specimens were aged for 1 h, we noted nano-sized Cu particles uniformly dispersed throughout the ferrite matrix. The precipitates were generally very small, with an average particle size of ~5.6 nm. With the increase of aging time, the dislocation density was significantly decreased in the sample subjected to the aging process for 3 h, as shown by the arrows in Figure 3e. The coarsening and growth of Cu precipitates were also observed in this case. As a result, the average equivalent diameter and Feret diameter of Cu precipitates gradually increased to ~16.1 and ~19.3 nm, respectively. Meanwhile, the morphology of Cu precipitates changed from spherical to ellipsoid in shape. Some Cu precipitates smaller than 10 nm were noted, indicating a continuous precipitation of Cu particles during further aging.

**Figure 4:**
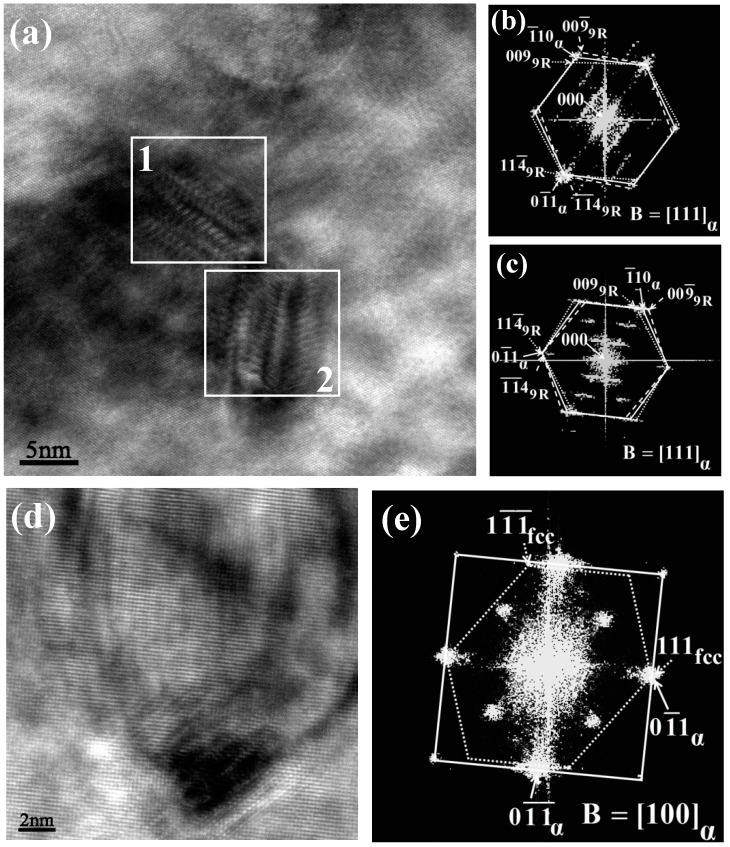
HRTEM images and their fast Fourier transform (FFT) patterns of Cu precipitates: (**a**) twinned 9R Cu image after aging for 1 h; (**b**) the FFT pattern of the square region 1 marked in (**a**); (**c**) the FFT pattern of the square region 2 marked in (**a**); (**d**) stable fcc Cu image after aging for 3 h; and (**e**) the FFT pattern of (**d**).

Figure 4 shows the HRTEM images and their FFT patterns of Cu precipitates. In the sample aged for 1 h, two precipitates with the size about 8 nm were found when observed along the [111] direction of the ferrite matrix. Typical twinned 9R Cu structure was identified from the corresponding FFT patterns, as evidenced by the observed (11-4)_9R_ twins and the (009)_9R_ closed-packed plane. The herringbone-type contrasts seen in the 9R Cu precipitates are believed to be important characteristics of the twinned 9R Cu structure [18,19,20]. In addition, g_003_, g_006_ and g_009_ spots could also be observed from the FFT patterns, indicating the stacking faults every third closed-packed plane. The precipitate–matrix orientation relationship obeyed [110]_9R_//[111]_α_, (11-4)_9R_//(0-11)_α_, as reported in previous studies [20,21,22].

It is theorized that the growth of the precipitates changes the crystal structure, as noted in the HRTEM images of a larger Cu precipitate (i.e., approximately 19 nm) in the sample aged for 3 h (Figure 4d), in which the beam direction is parallel to the direction of the [100] face in the ferrite matrix. As seen in the FFT pattern, the particle revealed a stable fcc Cu structure. The relationship between the precipitate and matrix could be described by the Kurdjumov–Sachs (K-S) orientation, [110]_fcc_//[11-1]_α_, (111)_fcc_//(0-11)_α_, which is consistent with previous studies [23,24].

It is widely accepted that Cu precipitates exhibit complex inner structures during the coarsening process. The formation of Cu precipitates starts with coherent bcc Cu structure [24,25]. These coherent bcc Cu precipitates cannot be observed in traditional TEM images due to the small difference in size between Cu and Fe atoms. According to Heo et al. [25], the bcc Cu precipitate can be observed using the subatomic-sized electron beam and a HADDF detector. In the HRTEM image, Cu columns exhibited brighter regions than iron columns, thus bcc Cu structure can be confirmed. The previous studies suggested that coherent bcc Cu structure directly transformed to incoherent fcc Cu structure during the growth of Cu precipitates. With the development of technology, Othen et al. [26,27] first proposed the transformation from bcc to 9R structure before the formation of fcc structure. They suggested that bcc Cu structure transformed to 9R Cu structure by a martensitic transformation. Subsequently, many authors confirmed the existence of 9R Cu structure. Researchers have noted that the transformation from bcc to 9R structure occurred when the Cu precipitates reached a critical size of 3–5 nm [22,25,28]. Feng et al. [29] found the coexistence of bcc and 9R orthogonal structure or 9R orthogonal and 9R monoclinic structure in a precipitate. It can be demonstrated that bcc Cu structure firstly transformed to 9R orthogonal structure, and then a transformation from 9R orthogonal to 9R monoclinic structure occurred. In general, 9R Cu structure is characterized by herringbone-type pattern and stacking faults, which could be observed in the HRTEM images with the beam direction parallel to the [111]_α_ direction. The fringe periodicity was 0.6 nm, which is equal to three times the close-packed plane spacing of 9R Cu structure. It is theorized that 3R Cu structure [26] or twinned fcc Cu structure [25] is formed from the 9R Cu structure during further aging. Finally, fcc Cu precipitates were formed, associated with coherency lost. Referring to the theories presented above, it can be concluded that, with an increase in the aging time, the precipitation and coarsening of Cu particles occurred in association with the evolution of crystal structure.

Due to the fast cooling rate, Cu atoms were presented in solid solution in the matrix in the water-cooled samples. During aging process, Cu precipitation occurred when both the aging temperature and time were at critical thresholds. Bcc Cu precipitates were first formed and then transformed to 9R structure during aging process [24,25]. 9R Cu precipitates could be observed during the 1-h aging experiment, indicating that transformation from bcc to 9R structure occurs at aging time less than 1 h. Formation of stable fcc Cu precipitates could be found when the aging time was 3 h.

### 3.3. Tensile Properties

**Figure 5:**
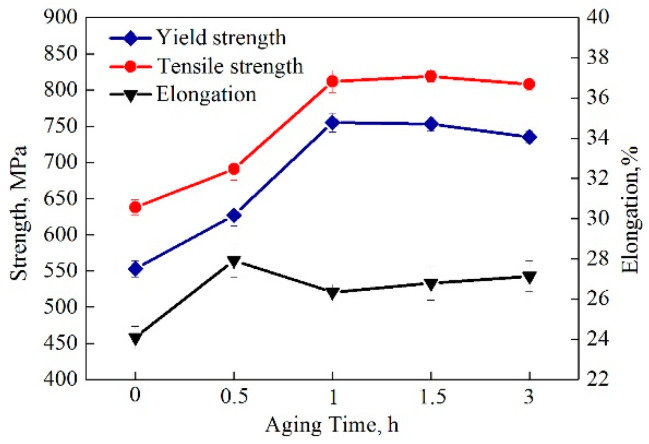
Variation of yield strength, tensile strength and elongation as a function of aging time.

Figure 5 shows the variations of tensile properties as a function of aging time. The yield strength, tensile strength and elongation all exhibited low values for the water-cooled samples. The corresponding values were 553 MPa, 638 MPa and 24.1%, respectively. Upon aging, all the steels achieved significant improvements on strength and ductility, which could be attributed to the synergistic effects of Cu precipitation strengthening and matrix softening. With the increase of aging time, the yield strength and tensile strength reached peak values after aging for 1 h. Beyond this point, these values decreased slowly, indicating the occurrence of an over-aging effect. The steel exhibited good tensile properties after being subjected to aging at 500 °C for 1 h, with a yield strength of 755 MPa, a tensile strength of 812 MPa and an elongation of 26.3%.

**Figure 6:**
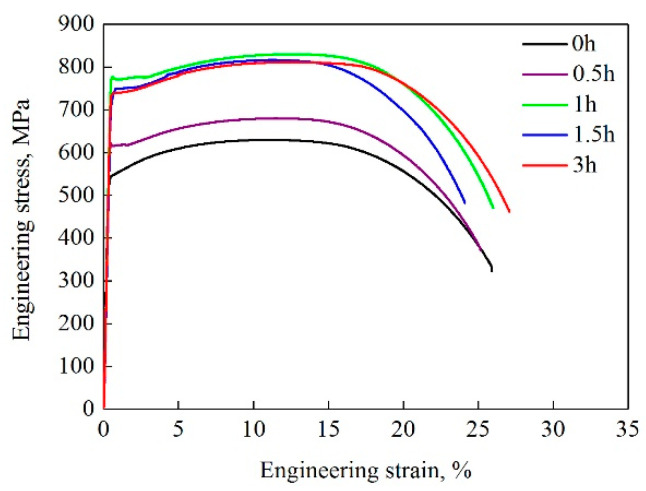
Tensile stress–strain curves of the steel samples subjected to different processing methods.

Figure 6 shows the tensile stress-strain curves obtained for the water-cooled and aged states. In the graph, it is clear that a short aging process (i.e., 0.5 h) only slightly improved the strength, as evidenced by the small increase of 74 MPa for the yield strength and 53 MPa for the tensile strength. Nevertheless, a strong strengthening effect could be observed in the samples aged for 1 and 3 h, as noted by the increase of 182–202 and 170–181 MPa in the yield and tensile strength values, respectively. In addition, yield plateaus could be observed in tensile stress–strain curves for the aged samples, whose generation could be attributed to the increased mobile dislocations during aging treatment.

The Cu precipitate–dislocation interaction strengthening effect can be calculated by use of the Russell–Brown model, as shown in Equation (1) [30,31]:

(1) $$\sigma_{PS}=M\frac{Gb}{L}{\left[1-{\left(\frac{E_{p}}{E_{m}}\right)}^{2}\right]}^{\frac{3}{4}};\;{sin}^{-1}\left(\frac{E_{p}}{E_{m}}\right)\geq 50^{\circ }$$

where $\sigma_{PS}$ represents Cu precipitate–dislocation interaction strengthening effect, *M* = 3 is the Taylor factor, *G* = 80 GPa is the shear modulus of the matrix, *b* = 0.25 nm is the Burgers vector of the dislocations and *E_p_* and *E_m_* represent the dislocation line energy in the Cu precipitates and matrix, respectively. L is the mean particle spacing in the slip plane, which can be calculated by Equation (2) [30,31]:

(2) $$L=0.866/{\left(RN\right)}^{1/2}$$

where *R* is the average radius of Cu precipitates and *N* is the average number density of Cu precipitates. In the present work, the thickness of the thin-film is considered to be 110 nm, thus *N* = 1.1 × 10^−^^5^/nm^3^ and 3.7 × 10^−^^6^/nm^3^ can be calculated for an aging time of 1 and 3 h, respectively. $\frac{E_{p}^{\infty }}{E_{m}^{\infty }}$ is given by Equation (3) [30,31]:

(3) $$\frac{E_{p}}{E_{m}}=\frac{E_{p}^{\infty }}{E_{m}^{\infty }}\frac{\log\frac{R}{r_{0}}}{\log\frac{r}{r_{0}}}+\frac{\log\frac{r}{R}}{\log\frac{r}{r_{0}}}$$

where $\frac{E_{p}^{\infty }}{E_{m}^{\infty }}$ = 0.62 represents the ratio of energy per unit length of a dislocation in the infinite media and *r* = 2.5 b and *r_0_* = 1000 *r* are defined as the inner and outer cut-off radii. Based on the Russell–Brown model, the Cu precipitate–dislocation interaction strengthening effects were calculated to be 232 and 199 MPa after aging at 500 °C for 1 and 3 h, respectively. According to the results of tensile tests, the yield strength of water-cooled steel was 553 MPa. The increments in yield strength were 202 and 182 MPa for an aging time of 1 and 3 h, respectively. From these findings, it can be concluded that the increment of yield strength was mainly governed by Cu precipitate–dislocation interaction strengthening during aging process.

It can be seen that the steel samples exhibited varying degrees of improvements in their strength parameters after the aging treatments, relative to the results seen in the water-cooled samples. Since Cu precipitation was difficult during the 0.5-h aging process, only a minuscule increase in the strength was noted. On the other hand, due to the appropriate size, distribution and number density of Cu precipitates, the most notable improvements were associated with the 1-h aging process. Continuous formation and coarsening of Cu precipitates during the 3-h aging process reduced the strength of the samples and led to over aging effect. In addition, the lowering of elongation could be attributed to the over strengthening of the matrix, which was caused by the large Cu precipitate–dislocation interaction strengthening effect with aging time over 0.5 h.

### 3.4. Low Temperature Impact Properties

**Figure 7:**
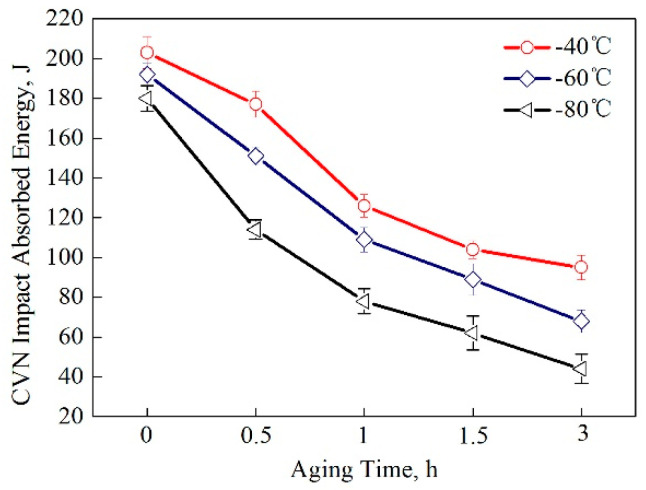
The Charpy V-notch (CVN) impact absorbed energies of the tested steel samples.

Figure 7 shows the variation of the CVN impact absorbed energies of the samples tested at −40, −60 and −80 °C as a function of the aging time. As noted, the water-cooled steel samples exhibited good low temperature toughness, with a CVN impact absorbed energy of 180 J at −80 °C. The CVN impact absorbed energy values gradually decrease with a longer aging process. The best results can be seen after the 1-h aging process, in which the CVN impact absorbed energy values obtained were 126, 109 and 78 J at −40, −60 and −80 °C, respectively. Since the aging process had an adverse effect on the low temperature toughness, it could be concluded that the improvement in the strength via aging treatments was accompanied by a decrease in the low temperature toughness.

**Figure 8:**
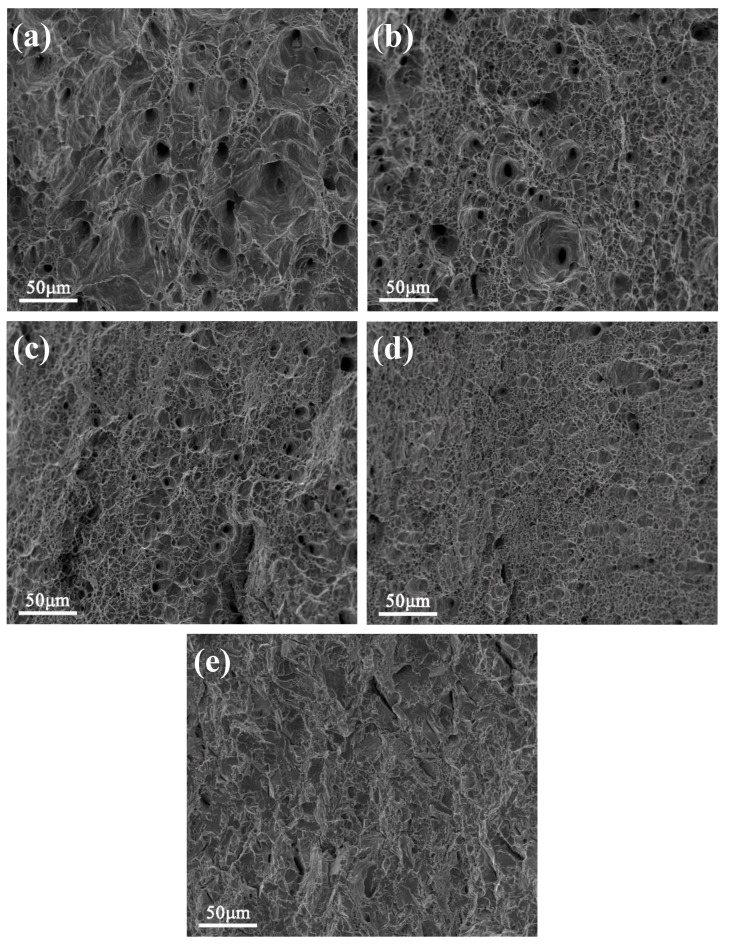
SEM micrographs showing the impact fracture surfaces at −80 °C after: (**a**) water cooling; and aging for (**b**) 0.5 h; (**c**) 1 h; (**d**) 1.5 h; and (**e**) 3 h.

Figure 8 shows the impact fracture surfaces of the tested steel samples. It can be seen that the fracture surfaces are distinctly different. Dimples in the fracture surfaces observed for the water-cooled samples and the samples aged for 0.5, 1 and 1.5 h were indicative of ductile fractures. The dimple size was found to be maximum for the water-cooled sample and minimum for the sample aged for 1.5 h. With a further increase in the aging time, the dimple size tended to become smaller. In addition, the fracture mode was brittle fracture for the sample aged for 3 h, as evidenced by the presence of quasi-cleavage nature. This observation was indicative of the deteriorating low temperature toughness of the samples subjected to prolonged aging. In the present work, Cu precipitation was the predominant factor that affected the low temperature toughness. It had a significant effect on strength enhancement while bringing a destruction to low temperature toughness simultaneously. The gradual decrease of CVN impact energy could be attributed to the formation and coarsening of Cu precipitates.

## 4. Conclusions

In this study, the aging process was applied to a Cu-bearing marine engineering steel. The relationship between microstructure and mechanical properties were discussed in detail. The major conclusions are as follows:

(1) The microstructure was characterized by polygonal ferrite and a small amount of bainitic ferrite in water-cooled condition. The structural recovery could be found during aging treatment, which became severe with the increase of aging time.
(2) With an increase in the aging time, the structural transformation from twinned 9R Cu to stable fcc Cu could be confirmed by HRTEM, together with the coarsening of Cu precipitates.
(3) Cu precipitate–dislocation interaction strengthening effects were calculated to be 232 and 199 MPa after aging at 500 °C for 1 and 3 h, respectively. The optimized samples could be obtained after a 1-h aging process where the yield strength, tensile strength and elongation showed high values of 755 MPa, 812 MPa and 26.3%, respectively.
(4) Conversely, the low temperature toughness was destroyed by prolonged aging, which was caused by Cu precipitation. The CVN impact energy values decreased continuously when the aging process was prolonged due to the formation and coarsening of Cu particles.

## Figures and Tables

**Table 1:** Chemical composition of the tested steel in mass %.

Fe	C	Si	Mn	P	S	Ni	Cr	Cu	Al	N
Bal.	0.005	0.2	0.7	0.01	0.004	2.5	0.5	2.0	0.03	0.005

## References

[1] Zhang Z.W., Liu C.T., Wen Y.R., Hirata A., Guo S., Chen G., Chen M.W., Chin B.A. (2012). Influence of aging and thermomechanical treatments on the mechanical properties of a nanocluster-strengthened ferritic steel. Metall. Mater. Trans. A.

[2] Das S., Ghosh A., Chatterjee S., Rao P.R. (2003). The effect of cooling rate on structure and properties of a HSLA forging. Scr. Mater..

[3] Ghosh A., Das S., Chatterjee S., Rao P.R. (2006). Effect of cooling rate on structure and properties of an ultra-low carbon HSLA-100 grade steel. Mater. Charact..

[4] Wen Y.R., Li Y.P., Hirata A., Zhang Y., Fujita T., Furuhara T., Liu C.T., Chiba A., Chen M.W. (2013). Synergistic alloying effect on microstructural evolution and mechanical properties of Cu precipitation-strengthened ferritic alloys. Acta Mater..

[5] Park T.W., Kang C.Y. (2000). The effects of PWHT on the toughness of weld HAZ in Cu-Containing HSLA-100 steel. ISIJ Int..

[6] Wei L.Y., Nelson T.W. (2012). Influence of heat input on post weld microstructure and mechanical properties of friction stir welded HSLA-65 steel. Mater. Sci. Eng. A.

[7] Momeni A., Arabi H., Rezaei A., Badri H., Abbasi S.M. (2011). Hot deformation behavior of austenite in HSLA-100 microalloyed steel. Mater. Sci. Eng. A.

[8] Deschamps A., Militzer M., Poole W.J. (2003). Comparison of precipitation kinetics and strengthening in an Fe-0.8%Cu alloy and a 0.8% Cu-containing low-carbon steel. ISIJ Int..

[9] Mulholland M.D., Seidman D.N. (2011). Nanoscale co-precipitation and mechanical properties of a high-strength low-carbon steel. Acta Mater..

[10] Isheim D., Gagliano M.S., Fine M.E., Seidman D.N. (2006). Interfacial segregation at Cu-rich precipitates in a high-strength low-carbon steel studied on a sub-nanometer scale. Acta Mater..

[11] Dhua S.K., Ray A., Sarma D.S. (2001). Effect of tempering temperatures on the mechanical properties and microstructures of HSLA-100 type copper-bearing steels. Mater. Sci. Eng. A.

[12] Hwang G.C., Lee S., Yoo J.Y., Choo W.Y. (1998). Effect of direct quenching on microstructure and mechanical properties of copper-bearing high-strength alloy steels. Mater. Sci. Eng. A.

[13] Ray P.K., Ganguly R.I., Panda A.K. (2003). Optimization of mechanical properties of an HSLA-100 steel through control of heat treatment variables. Mater. Sci. Eng. A.

[14] Ghosh A., Mishra B., Das S., Chatterjee S. (2004). An ultra low carbon Cu bearing steel: Influence of thermomechanical processing and aging heat treatment on structure and properties. Mater. Sci. Eng. A.

[15] Dhua S.K., Mukerjee D., Sarma D.S. (2003). Influence of thermomechanical treatments on the microstructure and mechanical properties of HSLA-100 steel plates. Metall. Mater. Trans. A.

[16] Panwar S., Goel D.B., Pandey O.P., Prasad K.S. (2003). Aging of a copper bearing HSLA-100 steel. Bull. Mater. Sci..

[17] Liu Q.D., Zhao S.J. (2013). Comparative study on austenite decomposition and Cu precipitation during continuous cooling transformation. Metall. Mater. Trans. A.

[18] Jung J.G., Jung M., Lee S.M., Shin E., Shin H.C., Lee Y.K. (2013). Cu precipitation kinetics during martensite tempering in a medium C steel. J. Alloys Compd..

[19] Wen Y.R., Hirata A., Zhang Z.W., Fujita T., Liu C.T., Jiang J.H., Chen M.W. (2013). Microstructure characterization of Cu-rich nanoprecipitates in a Fe-2.5 Cu-1.5 Mn-4.0 Ni-1.0 Al multicomponent ferritic alloy. Acta Mater..

[20] Monzen R., Iguchi M., Jenkins M.L. (2000). Structural changes of 9R copper precipitates in an aged Fe-Cu alloy. Philos. Mag. Lett..

[21] Bajguirani H.R.H., Jenkins M.L. (1996). High-resolution electron microscopy analysis of the structure of copper precipitates in a martensitic stainless steel of type PH 15-5. Philos. Mag. Lett..

[22] Monzen R., Jenkins M.L., Sutton A.P. (2000). The bcc-to-9R martensitic transformation of Cu precipitates and the relaxation process of elastic strains in an Fe-Cu alloy. Philos. Mag. A.

[23] Thompson S.W., Krauss G. (1996). Copper precipitation during continuous cooling and isothermal aging of A710-type steels. Mater. Trans. A.

[24] Lee T.H., Kim Y.O., Kim S.J. (2007). Crystallographic model for bcc-to-9R martensitic transformation of Cu precipitates in ferritic steel. Philos. Mag..

[25] Heo Y.U., Kim Y.K., Kim J.S., Kim J.K. (2013). Phase transformation of Cu precipitates from bcc to fcc in Fe-3Si-2Cu alloy. Acta Mater..

[26] Othen P.J., Jenkins M.L., Smith G.D.W. (1994). High-resolution electron microscopy studies of the structure of Cu precipitates in α-Fe. Philos. Mag. A.

[27] Othen P.J., Jenkins M.L., Smith G.D.W., Phythian W.J. (1991). Transmission electron microscope investigations of the structure of copper precipitates in thermally-aged Fe-Cu and Fe-Cu-Ni. Philos. Mag. Lett..

[28] Perez S.L., Jenkins M.L., Titchmarsh J.M. (2006). Evidence for deformation-induced transformations of Cu-rich precipitates in an aged FeCu alloy. Philos. Mag. Lett..

[29] Feng L., Zhou B.X., Peng J.C., Wang J.A. (2013). Crystal structure evolution of the Cu-rich nano precipitates from bcc to 9R in reactor pressure vessel model steel. Acta Metall. Sin. (Engl. Lett.).

[30] Russel K.C., Brown L.M. (1972). A dispersion strengthening model based on differing elastic moduli applied to the iron-copper system. Acta Metall..

[31] Kong H.J., Xu C., Bu C.C., Da C., Luan J.H., Jiao Z.B., Chen G., Liu C.T. (2019). Hardening mechanisms and impact toughening of a high-strength steel containing low Ni and Cu additions. Acta Mater..

